# Paraspinal muscle degeneration and lower bone mineral density as predictors of proximal junctional kyphosis in elderly patients with degenerative spinal diseases: a propensity score matched case–control analysis

**DOI:** 10.1186/s12891-022-05960-z

**Published:** 2022-11-23

**Authors:** Tong-tong Zhang, Jun-zhe Ding, Chao Kong, Wei-guo Zhu, Shuai-kang Wang, Shi-bao Lu

**Affiliations:** Xuanwu Hospital of Capital Medical University, Beijing, China

**Keywords:** Proximal junctional kyphosis, Degenerative spine disease, Paraspinal muscle, Bone mineral density, Propensity score matching

## Abstract

**Study design:**

Retrospective case–control study.

**Objectives:**

Proximal junctional kyphosis (PJK) is a postoperative complication involving the proximal segments which is commonly seen in patients with degenerative spine diseases (DSD). The purpose of the present study was to identify predictive factors for postoperative PJK in elderly patients with DSD.

**Methods:**

We reviewed elderly patients with DSD who underwent thoracolumbar fusion involving no less than 3 levels. Patients who developed PJK were propensity score-matched with patients with DSD who received the same procedure but did not develop PJK. Demographic characteristics, sagittal vertical axis (SVA), computed tomography (CT) value (Hounsfield unit), and paraspinal muscle parameters were compared between PJK and non-PJK groups.

**Results:**

Eighty-three PJK and non-PJK patients were selected by propensity score matching for age, sex, history of smoking, body mass index, number of fused segments, and upper instrumented vertebra (UIV) location. SVA showed no significant difference between the two groups. In PJK group, fatty infiltration (FI) in erector spinae and multifidus was significantly greater, while the relative cross-sectional area (rCSA) of erector spinae was significantly smaller than that in non-PJK group. CT value was significantly lower in PJK group. Lower erector spinae rCSA and CT value of the UIV, higher erector spinae FI and multifidus FI were identified as predictors of postoperative PJK.

**Conclusions:**

PJK is a common complication in older patients with DSD. Paraspinal muscle degeneration and low bone mineral density of the UIV are predictors of PJK. Protective measures targeting paraspinal muscles and the UIV may help prevent postoperative PJK.

**Supplementary Information:**

The online version contains supplementary material available at 10.1186/s12891-022-05960-z.

## Background

Population ageing is a global phenomenon that is attributable to the progressive increase in life expectancy over the last few decades. Elderly patients present a range of challenges for clinicians. In our spinal surgery practice, we have noticed an increasing number of patients with degenerative spine diseases (DSD) who opt for surgical intervention rather than conservative treatment due to longer life expectancy and improved surgical outcomes. Elderly patients with DSD are at a higher risk of complications, complicated comorbidities, and lower compensatory capacity. As elderly patients tend to have reduced bone mineral density (BMD) and greater degree of spinal imbalance, postoperative mechanical complications have become a major concern [1]. Proximal junctional kyphosis (PJK) is a postoperative complication involving the proximal segments that is commonly seen in surgically-treated DSD cases [2]. Glattes et al. first defined PJK as a proximal junctional sagittal Cobb angle between the lower endplate of upper instrumented vertebra (UIV) and the upper endplate of 2 supra-adjacent vertebrae ≥ 10° and at least 10° greater than the preoperative measurement [3]. PJK results in poor surgical outcomes due to pain, deformity, instability, disability, and potential neurologic deficits. The reported incidence of PJK ranges from 5 to 46%, with two-thirds of cases occurring within 3 months after surgery and 80% of cases occurring within 18 months after surgery [4].

Of late, much attention has been paid to prevent PJK and achieve satisfactory outcomes. Previous studies indicated that age, BMD, numbers of fused segments, and UIV location are risk factors for postoperative PJK in patients with DSD [4–13]. Recently the role of paraspinal muscles in the process of spinal stability and degenerative changes is increasingly being recognized. As a spinal stabilizer and an effector for maintaining sagittal balance, the function of the paraspinal muscles affects the risk of postoperative mechanical complications [4, 14]. However, PJK in elderly DSD patients is a multi-factorial postoperative complication. A variety of factors, including age-related comorbidities and degenerative changes, surgical approach and procedures makes it difficult to distinguish the effects of anatomic factors on UIV. For elderly patients with long segments fusion, the influence of anatomic factors on the occurrence of postoperative PJK remains unclear. Therefore, we aimed to identify predictive factors for postoperative PJK in elderly patients with DSD. We hypothesized that elderly DSD patients with paraspinal muscle degeneration and reduced BMD at UIV have a higher incidence of postoperative PJK.

## Methods

### Patients

This study was approved by the Ethics Committee of the Xuanwu hospital, Capital Medical University. The patient hospitalization number were used to encode demographic information and surgical data of the subjects. All parties were fully aware of the confidentiality requirements under the Helsinki Declaration. The requirement for written informed consent of patients was waived off by the Ethics Committee of Xuanwu hospital as this was a retrospective study.

We retrospectively reviewed elderly patients (age > 65 years) with DSD who were diagnosed and treated at our center between January 2016 and December 2019. Patients with DSD who underwent thoracolumbar fusion with fusion of no less than 3 levels were eligible for inclusion. The exclusion criteria were: neuromuscular diseases, spinal infection, ankylosing spondylitis, spinal tumor, and previous spinal trauma or surgery. We also excluded patients who underwent minimally invasive lumbar fusion surgery and patients with cement-augmented pedicle screws.

Radiological PJK was defined as a sagittal Cobb angle between the UIV and the two levels above the UIV (UIV + 2) of ≥ 10° and at least 10° greater than the preoperative measurement [2]. According to the diagnostic criteria, a total of 108 patients with postoperative PJK were screened out. As a control group, 225 DSD patients who received the same procedure without PJK were selected by propensity score matching for age, sex, history of smoking, BMI, number of fused segments, and UIV location (lower thoracic spine or upper lumbar spine).

The image data of all patients were collected and measured using the hospital's built-in Picture Archiving and Communication System (PACS). All patients underwent preoperative full spine standing x-ray, computed tomography (CT), and magnetic resonance imaging (MRI) of the corresponding spinal area. Postoperative full spine standing x-ray was obtained during the follow-up period at the time points of 1, 3, 6, 12, 24, and 36 months. A minimum of 18-month follow-up was required.

### Surgical procedure

Posterior surgery consisted of a standard posterior midline approach with implementation of a bilateral pedicle screws and rods system. Decompression of the spine was then carried out using laminectomy or foraminotomy with complete decompression of the central canal and the lateral recesses. Laminae, transverse processes, and facet joints were thoroughly decorticated to expose adequate bleeding bony surfaces for interlaminar and intertransverse fusion.

## Parameters

**Fig. 1:**
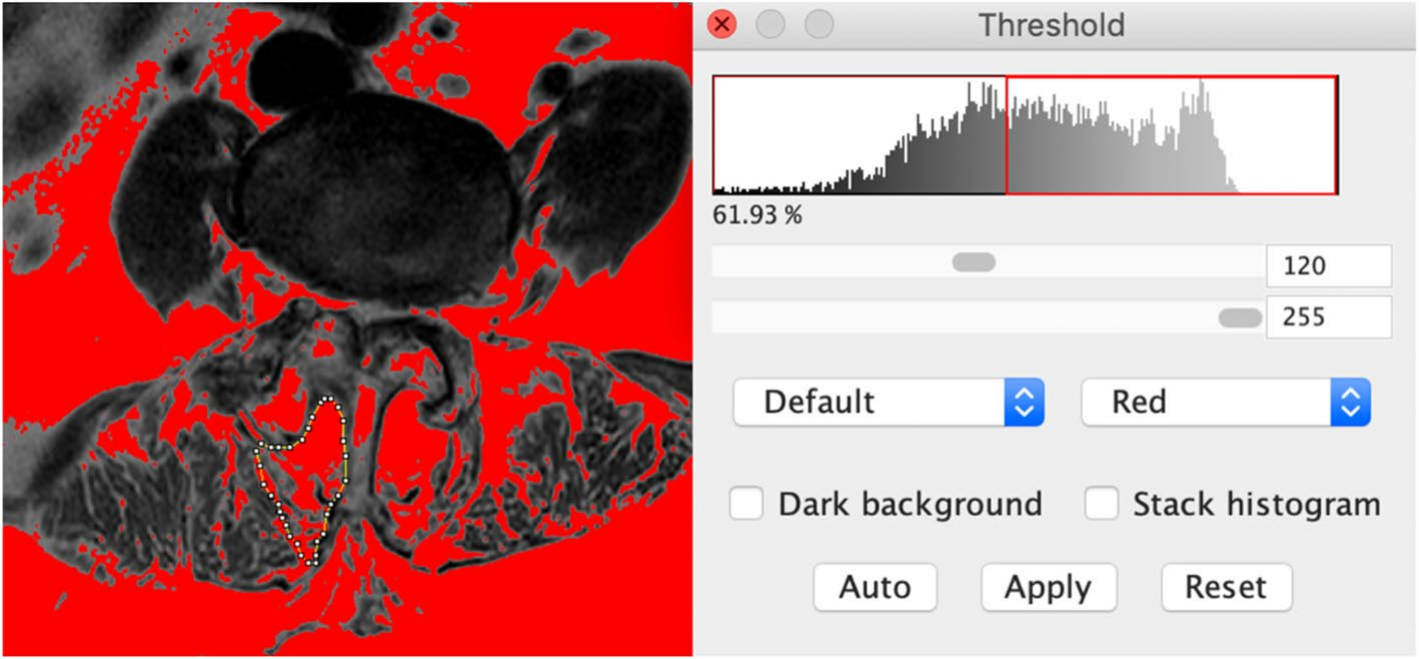
Measurement method of the multifidus muscle fatty infiltration. The Image J Threshold method on the middle layer of magnetic resonance image in each segment

The paraspinal muscle cross-sectional area (CSA) measurement was made in the middle layer of the MRI on L1/L2 segment using the gray-scale discrimination method proposed by Ranson et al. [15]. CSA of erector spinae (ES) and multifidus muscle (MF) was obtained by dividing the region of interest (ROI) according to the boundaries of each paraspinal muscle on the cross section. Relative cross-sectional area (rCSA) was adopted to eliminate the individual differences in muscle volume that may affect the results [16]. rCSA is the ratio of the paraspinal muscle CSA to the CSA of the vertebra body of the same segment. The mean value of both sides was measured and adopted for analysis. To measure the degree of fatty infiltration (FI) in paraspinal muscles, we defined the middle layer of the MR image as the measurement plane of the segment, and used the threshold method on Image J (National Institutes of Health, Bethesda, MD, USA). Namely, the percentage of the number of fat pixels in the total number of pixels in each paraspinal muscle ROI [17]. A threshold gray-scale value of 120 was used to distinguish the pixels of intramuscular fatty tissue [18]. The measurement method is illustrated in Fig. 1. Sagittal vertical axis (SVA) was measured on preoperative full spine standing x-ray.

The CT HU value of upper instrumented vertebra was measured on preoperative thoracic/lumbar CT scans by dividing the ROI on three layers of axial images of the vertebra body. Average HU value of three ROIs was used to represent the BMD of the UIV [19, 20].

All parameters were separately measured by two orthopedic surgeons with specialized training in radiographic measurement. The mean value of the two measurements was adopted for analysis.

### Statistical analysis

Statistical analysis was conducted using SPSS 26.0 (IBM Corp., USA). Continuous variables are presented as mean ± standard deviation. Parameters of PJK patients were compared with the propensity-score matched control group using student *t* test and Chi-squared test. Receiver operating characteristic (ROC) curves were constructed for SVA, paraspinal muscles parameters, and CT HU value of the UIV. The optimal cutoff values to differentiate between patients with PJK and control patients were determined. Intra-observer reliability and the inter-observer reliability were evaluated using the intraclass correlation coefficient (ICC) [21]. ICCs less than ± 0.40 indicate poor, ± 0.40–0.75 indicate fair or good, and ± 0.75–1.00 indicate excellent reliability [22]. *P* values < 0.05 were considered indicative of statistical significance.

## Results

### Patient demographics

A total of 1832 patients undergoing posterior lumbar fusion were examined, of which 333 patients qualified the inclusion criteria and had obtained sufficient follow-up. The prevalence of PJK was 32.4% (*n* = 108) and the average follow-up was 24.2 months (range 18–46). Among them, 83 PJK and non-PJK patients were selected by propensity score matching for age, sex, history of smoking, BMI, number of fused segments, and UIV location. Unmatched and matched parameters are summarized in Tables 1 and 2.

**Table 1:** Patient demographics of unmatched PJK and Non-PJK group

	PJK	Non-PJK	*P*-Values
Number of patients	108	225	
Age (yrs.)	74.0 ± 6.0	70.6 ± 4.2	< 0.001
Female	74	145	0.463
History of smoking	18	28	0.296
BMI	27.58 ± 3.72	25.73 ± 3.53	< 0.001
Levels fused	4.3 ± 1.7	3.8 ± 1.3	0.007
UIV			
Thoracic	34	52	
Lumbar	74	173	0.102

*BMI* body mass index, *UIV* upper instrumented vertebra. All values are expressed as mean value ± standard deviation

**Table 2:** Patient demographics of PJK and Non-PJK group after propensity score matching

	PJK	Non-PJK	*P*-Values
Number of patients	83	83	
Age (yrs.)	72.5 ± 5.6	72.6 ± 4.8	0.953
Female	56	59	0.614
History of smoking	14	12	0.669
BMI	27.36 ± 3.82	27.19 ± 3.36	0.763
Levels fused	4.0 ± 1.5	4.2 ± 1.6	0.513
UIV			
Thoracic	22	25	
Lumbar	61	58	0.605

*BMI* body mass index, *UIV* upper instrumented vertebra. All values are expressed as mean value ± standard deviation

### Preoperative parameters

We compared preoperative SVA, CT HU value, and muscle parameters between PJK patients and propensity score matched cohort of non-PJK patients. SVA showed no significant difference between the two groups. Paraspinal muscle parameters were found significantly different between PJK and non-PJK patients. ES and MF FI of PJK patients were significantly greater, while the ES rCSA was significantly smaller compared to the non-PJK group. CT HU value of the UIV was significantly lower in the PJK group. On combining the rCSA of ES and MF, the PJK group showed significantly smaller extensor muscle rCSA compared to non-PJK patients. There was excellent intra-observer and inter-observer reliability with respect to measurements for muscle rCSA and FI (ICCs > 0.8). The preoperative parameters are summarized in Table 3.

**Table 3:** Comparison of preoperative parameters between PJK and Non-PJK patients

	PJK	Non-PJK	*P*-Values
Number of patients	83	83	
SVA	5.53 ± 3.38	5.70 ± 3.16	0.729
CT-HU	107.07 ± 30.62	123.28 ± 35.59	0.002
rCSA-ES	12.16 ± 4.19	14.17 ± 5.97	0.013
rCSA-MF	3.39 ± 1.32	3.61 ± 2.20	0.430
rCSA-ES + MF	15.18 ± 3.98	17.65 ± 5.81	< 0.001
FI-ES	47.6 ± 10.4%	43.4 ± 8.7%	0.006
FI-MF	59.4 ± 6.9%	54.0 ± 6.4%	< 0.001

*SVA* sagittal vertical axis, *HU* hounsfield unit, *rCSA* relative paraspinal muscle cross area, *FI* fatty infiltration, *MF* multifidus muscle, *ES* erector spinae. All values are expressed as mean value ± standard deviation

### Predictive factors

**Fig. 2:**
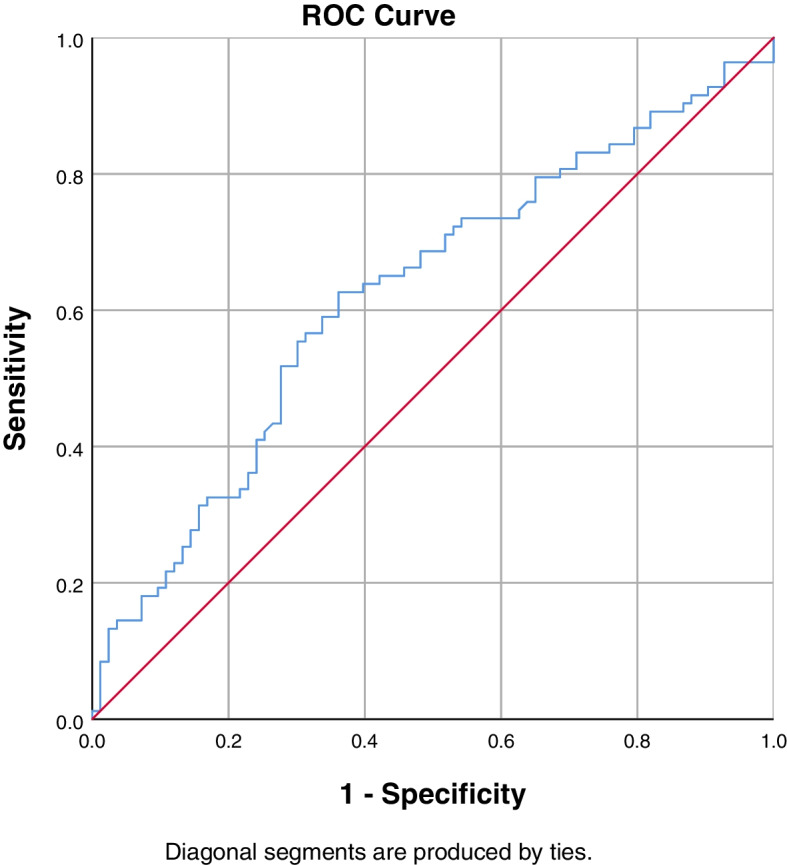
ROC curve to find the optimum cut-off point of ES rCSA to predict postoperative PJK. AUC = 0.624 (95% CI, 0.539–0.709)

**Fig. 3:**
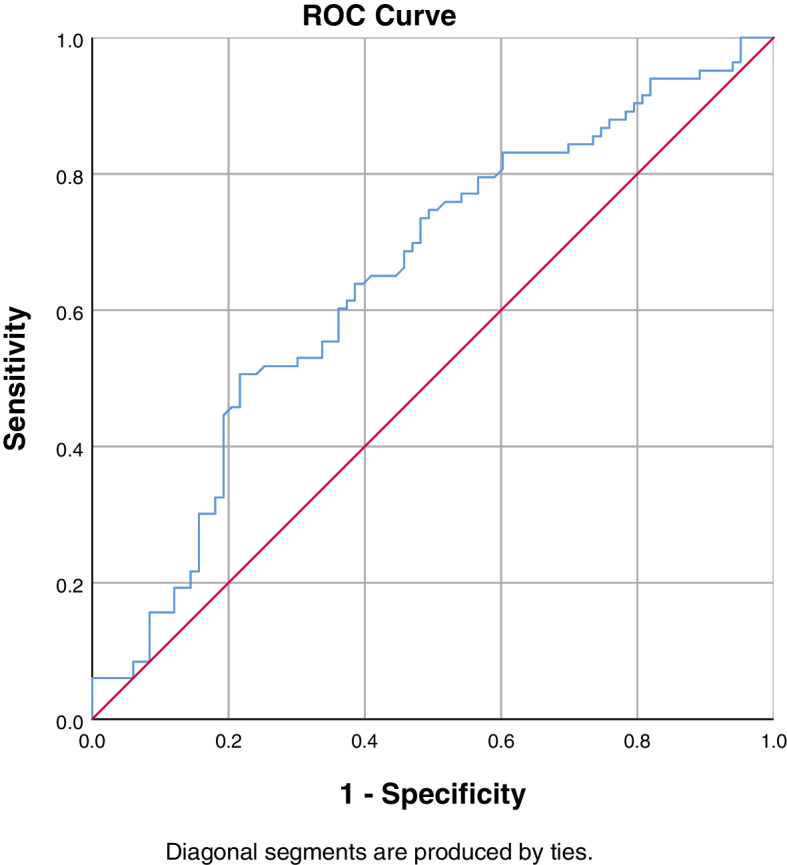
ROC curve to find the optimum cut-off point of CT HU value of the UIV to predict postoperative PJK. AUC = 0.646 (95% CI, 0.562–0.730)

**Fig. 4:**
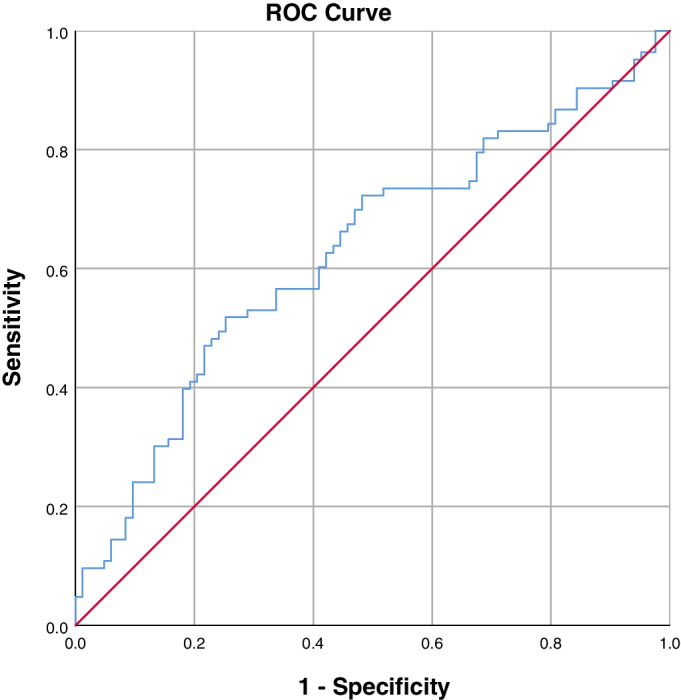
ROC curve to find the optimum cut-off point of ES FI to predict postoperative PJK. AUC = 0.628 (95% CI, 0.542–0.713)

**Fig. 5:**
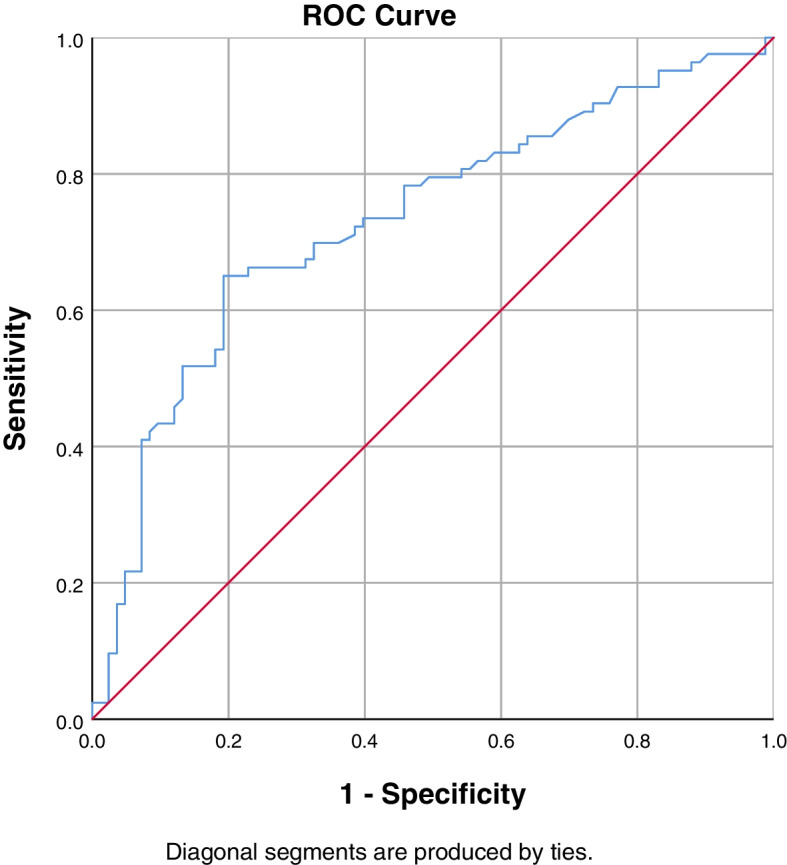
ROC curve to find the optimum cut-off point of MF FI to predict postoperative PJK. AUC = 0.732 (95% CI, 0.654–0.809)

On ROC curve analysis, the optimal ES rCSA cut-off value of 12.17 cm^2^ was associated with 62.7% sensitivity and 63.9% specificity for the diagnosis of PJK [AUC: 0.624 (95% CI, 0.539–0.709)]. The optimal cut-off value of CT HU value of UIV was 120.87, which was associated with 50.6% sensitivity and 78.3% specificity for the diagnosis of PJK [AUC: 0.646 (95% CI, 0.562–0.730)]. The optimal ES FI cut-off value of 47.90% was associated with 51.8% sensitivity and 74.7% specificity for the diagnosis of PJK [AUC: 0.628 (95% CI, 0.542–0.713)]. The optimal MF FI cut-off value of 58.03% was associated with 65.1% sensitivity and 80.7% specificity for the diagnosis of PJK [AUC: 0.732 (95% CI, 0.654–0.809)]. The ROC curves of the above-mentioned parameters are presented in Figs. 2,3,4, and 5.

## Discussion

**Fig. 6:**
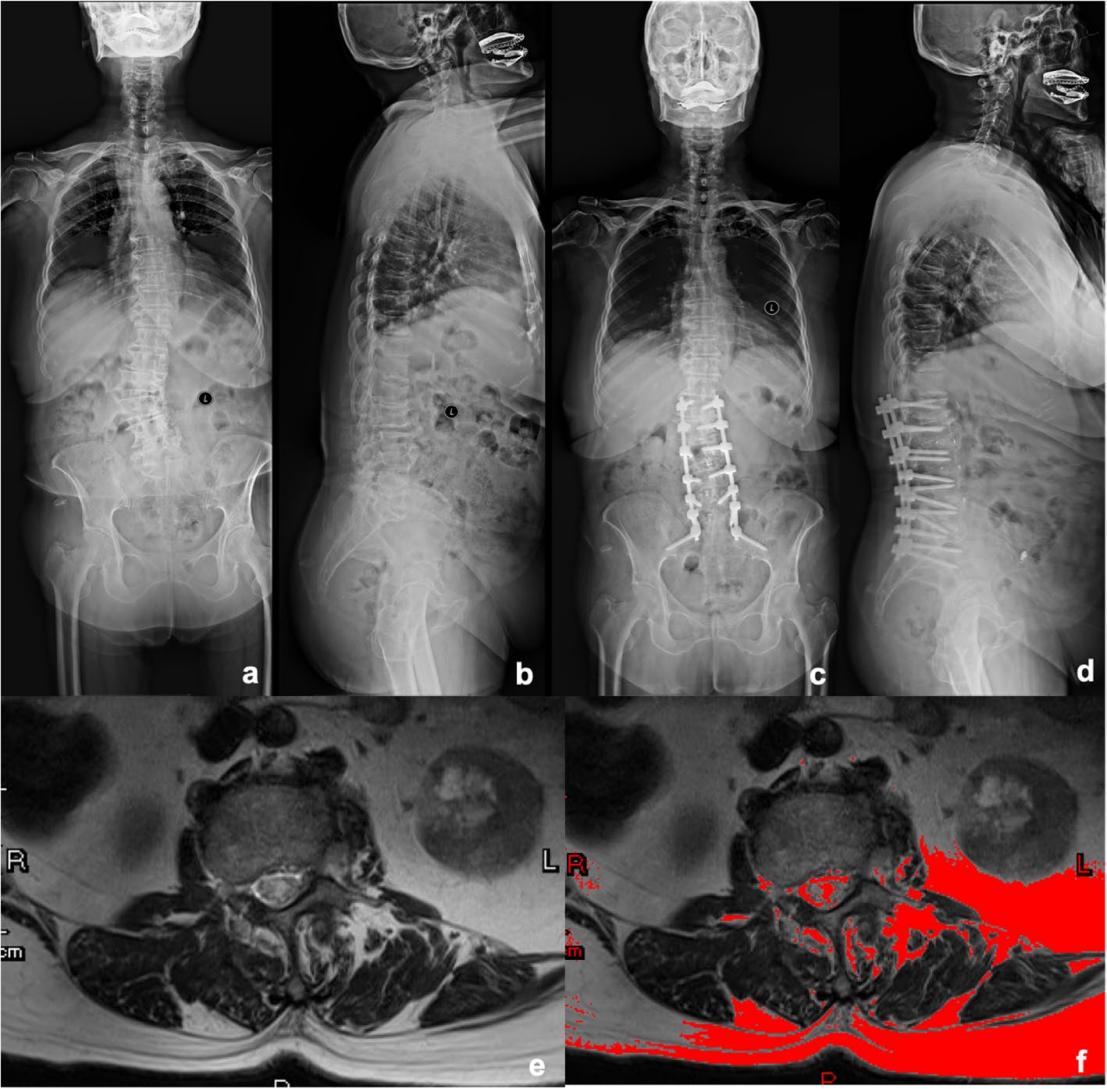
A 78-year-old woman with a diagnosis of degenerative spinal deformity. Posterior decompression and posterior instrumentation with pedicle screw fixation from L1-S2. Preoperative proximal junctional angle as 2.2 degree. Last follow-up proximal junctional angle as 13.6 degree

**Fig. 7:**
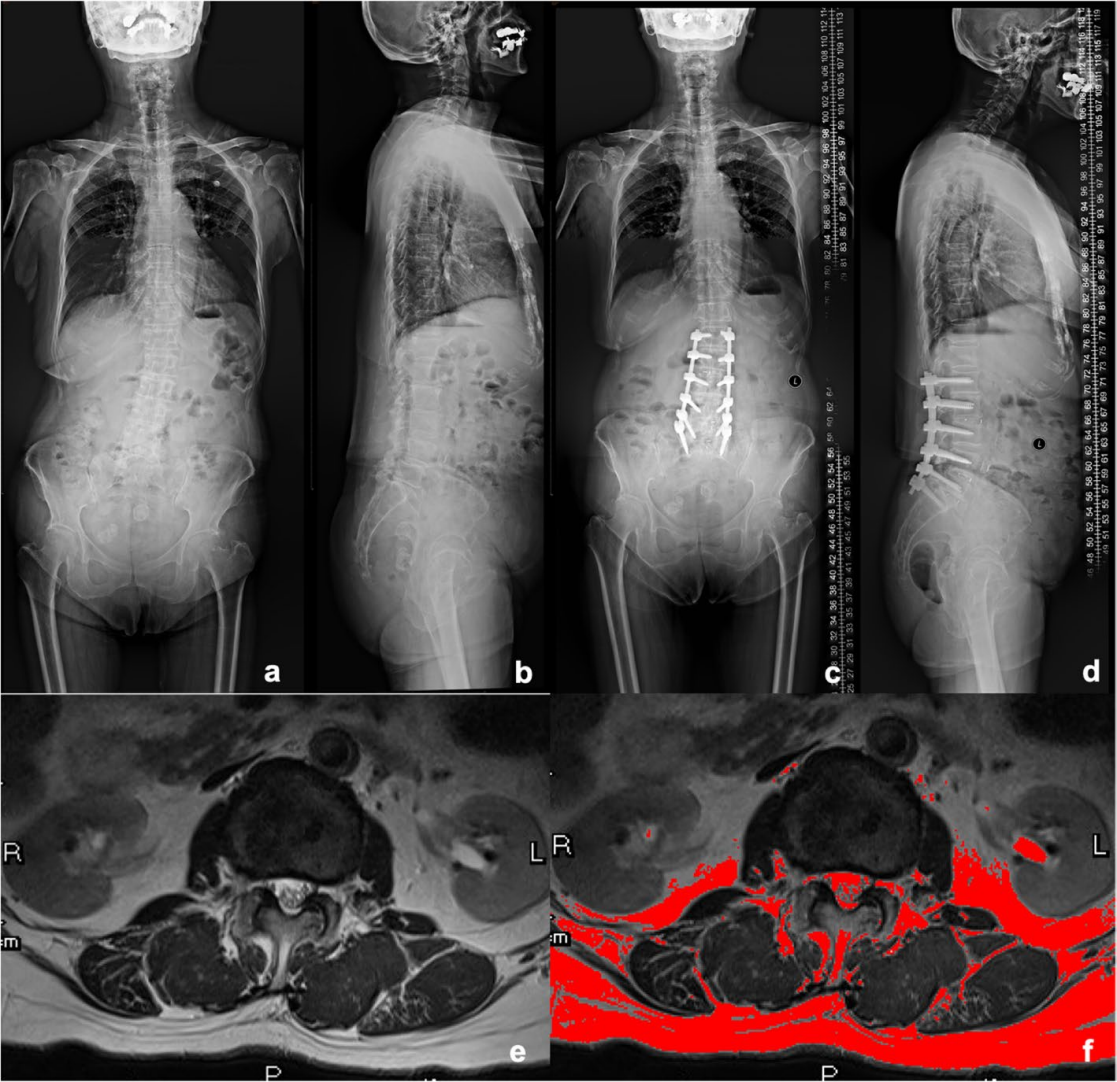
An 82-year-old woman with a diagnosis of degenerative lumbar spondylolisthesis. Posterior decompression and posterior instrumentation with pedicle screw fixation from L1-S1. Preoperative proximal junctional angle as 0.4 degree. Last follow-up proximal junctional angle as 3.1 degree

PJK is a commonly encountered complication of internal fixation after surgical intervention for DSDs. Several risk factors for PJK have been identified in the contemporary literature [4, 6–8, 23]. In the present study, greater FI of MF and ES, lower rCSA of ES and CT HU value of the UIV were related to postoperative PJK, with cut-off values of 58.03%, 47.90%, 12.17 cm^2^, and 120.87, respectively. Typical cases of PJK and non-PJK group were presented in Figs. 6 and 7.

Surgical, radiological and patient-related factors have all been implicated in the occurrence of PJK [9–11, 24–28]. Greater age at surgery and larger BMI are proven risk factors for PJK [6, 11, 23, 28]. Compared to younger individuals, elderly patients often have lower BMD, lesser muscle mass, and may require more aggressive spinal correction. These factors make elderly patients vulnerable to postoperative PJK. Therefore, spine surgeons should pay special attention to the prevention of PJK in elderly patients. Some instrumentation techniques such as hook or hybrid instrumentation may lead to lower incidence of PJK compared to the use of pedicle screws in the upper instrumented level [24, 29–31]. In the present study, pedicle screw fixation was applied in all cases by the same surgical team. Many studies have shown that the selection of UIV may affect the incidence of PJK [6, 12, 13, 32]. By conducting propensity score matching of cases, the present study aimed to minimize the impact of age, sex, BMI, and surgical factors on the incidence of PJK.

The mechanism of PJK can be divided into osseous failure of the vertebrae and posterior ligamentous complex failure. As a subtype of PJK, Hart et al. initially defined proximal junctional failure (PJF) as acute fracture and collapse of proximal junctional structure [33]. Yagi et al. divided PJK into 3 types: PJK from disc and ligamentous failure as type 1, bone failure as type 2, implant/bone interface failure as type 3 [25]. Their first type corresponds to posterior ligamentous complex failure and the second and third types are vertebrae osseous failure. Therefore, the predictive factors and preventive measures of PJK can be summarized into these two major categories.

The present study demonstrated the relation between degeneration of paraspinal muscles and PJK. PJK patient group showed lower rCSA and higher FI of both MF and ES. These result are consistent with those of previous studies [4, 27, 34]. Paraspinal muscle degeneration presents as atrophy and fatty infiltration [35, 36]. In the present study, greater FI of MF and ES, and lower rCSA of ES were related to postoperative PJK. These results indicated that paraspinal muscle degeneration is an independent risk factor for postoperative PJK. As an important stabilizer of the spine, the paraspinal muscles play an important role in the degenerative process of the spine. Fully functional paraspinal muscles help maintain the stability of the spine and slow the progression of spinal degeneration. Several recent studies have shown an equally important role of the paraspinal muscles as a stabilizer after spinal fusion [14, 27, 34]. Functional paraspinal muscles can provide protection and reduce the mechanical stress on the proximal segments, thereby reducing the risk of postoperative PJK [27]. Paraspinal muscle degeneration is commonly seen in DSD patients. Therefore, paraspinal muscle assessment should be incorporated into routine preoperative planning.

Moreover, surgical exposure of the spine can also cause damage to the paraspinal muscles. Mechanical instability caused by soft-tissue disruption is concentrated at the proximal junction, causing damage to the UIV and adjacent segments [37]. At the same time, postoperative PJK disrupts the integrity and stability of the proximal junctional biomechanical structure. These changes may lead to further paraspinal muscle degeneration. Therefore postoperative paraspinal muscles degeneration and PJK can be mutually causal, and further longitudinal studies are required to clarify the causal relationship. For DSD patients with severe paraspinal muscle degeneration, compensatory protective measures should be implemented with respect to the selection of UIV, the type of internal fixation, and the enhancement techniques. Francisco et al. introduced a new strap enhancement technique applied in posterior spinal fusion which showed a protective effect against PJK [38]. Intra-operative measures such as conscious preservation of the paraspinal muscles and augmentation of the posterior ligamentous complex have also been shown to prevent PJK.

The results of the present study showed that lumbar spine BMD (CT HU value of lumbar vertebrae) is related to the occurrence of PJK. The CT value of vertebrae in PJK group was significantly lower than those in the non-PJK group. The bone quality of the proximal segments, especially the UIV and UIV + 1, is an important determinant of the stability of the internal fixation including pedicle screws [39, 40]. In recent studies, lower Hounsfield units at the UIV and UIV + 1 showed a significant association with PJK and PJF [41, 42]. Degenerative bone mass loss is manifested as osteopenia and osteoporosis, which are commonly seen in DSD patients [43, 44]. These conditions should be thoroughly assessed and considered during the preoperative planning process. Recently, several preventive measures have been proposed to reduce the risk of PJK. The use of bisphosphonates or biosynthetic parathyroid hormone in combination with calcium and vitamin D is effective in maintaining bone mass in elderly patients [45]. Regular anti-osteoporosis therapy may have important implications for preventing fixation-related complications including PJK, especially in elderly patients. In addition, augmentation techniques such as vertebroplasty at UIV and UIV + 1 have been shown to be a protective factor [46].

Targeted surgical strategies and techniques need to be applied in elderly patients to obtain satisfactory clinical outcomes. Owing to the progressive population aging, elderly patients account for an increasing proportion of the DSD patient population. Surgical intervention in elderly DSD patients poses complex challenges. Elderly DSD patients are at higher risk of developing paraspinal muscle degeneration and osteoporosis than younger patients. The presence of these degenerative factors increases the risk of mechanical complications, including PJK. Previous studies have suggested that surgical intervention in elderly DSD patients requires age-based strategies including preoperative planning, the use of “soft landing” with hooks instead of pedicle screws at the proximal end of the construct, cement augmentation in UIV and UIV + 1, posterior ligamentous complex reinforcement with polyethelyene tether, and protection of paraspinal muscles [12, 30, 46, 47].

Some limitations of our study should be considered while interpreting the results. First, measurement errors in the manual selection of the ROI of the paraspinal muscles cannot be ruled out. However, we minimized the scope for errors by measuring paraspinal FI using a grayscale threshold method. In addition, the measurements were independently performed by two specifically trained orthopedic surgeons, and the mean value of the two measurements was used for analysis. Second, since not all DSD patients had undergone thoracic MRI, we selected L1/L2 level for paraspinal muscle measurements. Although the paraspinal muscle degeneration tends to be consistent among different levels, the L1/L2 level parameters may not be entirely representative of the condition of paraspinal muscles in other UIV segments [48]. Future studies should include assessment of the entire lumbar paraspinal muscles. Lastly, we measured muscle parameters including rCSA and FI to assess muscle degeneration. However, there is no clear consensus whether radiological parameters are sufficiently representative of muscle function [35, 36]. Use of electromyography and muscle strength measurements can help provide more robust evaluation.

## Conclusions

PJK is a common complication in older patients with DSD. Paraspinal muscle degeneration and low bone mineral density of the UIV are predictors of PJK. Protective measures targeting paraspinal muscles and the UIV may play a key role in preventing postoperative PJK.

## Supplementary Information

**Additional file 1.**

## Data Availability

The datasets used and/or analysed during the current study available from the corresponding author on reasonable request.

## Abbreviations

DSD: Degenerative spine diseases
BMD: Bone mineral density
PJK: Proximal junctional kyphosis
UIV: Upper instrumented vertebra
CSA: Cross-sectional area
ES: Erector spinae
MF: Multifidus muscle
ROI: Region of interest
rCSA: Relative cross-sectional area
FI: Fatty infiltration
SVA: Sagittal vertical axis
ICC: Intraclass correlation coefficient
PJF: Proximal junctional failure
